# The COVID-19 Severity and Its Association with Intestinal Parasite Coinfection and Urine Biochemical Parameters among COVID-19-Confirmed Patients Admitted to Debre Markos University COVID-19 Center, Northwest Ethiopia

**DOI:** 10.1155/2024/3064374

**Published:** 2024-01-13

**Authors:** Milkiyas Toru, Aytenew Atnaf, Hylemariam Mihiretie Mengist, Alemayehu Reta

**Affiliations:** Department of Medical Laboratory Science, College of Health Science, Debre Markos University, Debre Markos, Ethiopia

## Abstract

**Background:**

Though most people with COVID-19 disease show asymptomatic to mild illness, a substantial number of patients are at high risk of developing severe disease and adverse outcomes with long COVID-19 and death. Even though some studies showed that previously existing infections with parasites amend the host's body defenses to increase resistance to infection with SARS-CoV-2, there is limited data in Ethiopia.

**Objectives:**

This study is aimed at determining the COVID-19 disease severity and its association with intestinal parasite coinfection and urine biochemical parameters among COVID-19-confirmed patients admitted at Debre Markos University COVID-19 Center, 2021.

**Methods:**

A prospective cohort study was conducted on 136 RT-qPCR-confirmed COVID-19 patients admitted at Debre Markos University COVID-19 Center from January 1 to March 30, 2021. Sociodemographic and clinical data were collected by using standardized data collection forms. A urine biochemical test was performed using a dry urine dipstick kit and stool examination using direct wet mount microscopic examination and formalin-ether concentration method. The chi-square test, Fisher exact test, and ordinal logistic regression analysis were computed to assess association with outcome variables using Statistical Package for Social Science software (version 24).

**Result:**

A total of 136 COVID-19-confirmed patients participated in this study. The median age of the participants was 48 years. The majority (86 (62.5%)) of them were male in sex. Of the 136 cases, 39 (28.7%) had died. Among the 136 patients, 22 (16.2%) were coinfected with intestinal parasites. COVID-19 patients who have intestinal parasite coinfection had lower odds of developing clinically severe COVID-19 compared to noninfected (AOR = 0.37; 95% CI = 0.147‐0.944; *P* = 0.037). The majority (104 (76.5%)) of them have abnormal urine biochemical results. From the abnormal urine biochemical tests observed, the urine blood, glucose, and ketone tests were positive for 54 (39.7%), 36 (26.5%), and 30 (21.1%) patients, respectively. Among the 31 critical COVID-19 patients, 25 (80.6%) showed abnormal urine biochemical parameters. Age and comorbidity were significantly associated with COVID-19 severity (*P* < 0.05).

**Conclusion:**

Patients with old age and comorbidity had an increased risk of developing severe COVID-19 disease. Patients having SARS-CoV-2 and intestinal parasitic coinfections demonstrated mild COVID-19 disease severity. Abnormal urine biochemical results were common among critical COVID-19 patients. Thus, advanced study on the effect of the interaction among intestinal parasites on COVID-19 clinical severity and its mechanisms is essential.

## 1. Background

Serious acute pneumonia was an outbreak in Wuhan, China, in December 2019. Since then, the virus has been attacking the whole world. By deep sequencing analysis of specimens obtained from the lower respiratory tract and throat swabs, a novel coronavirus was identified on Jan 7, 2020. The virus was named by the World Health Organization (WHO) as severe acute respiratory syndrome coronavirus 2 (SARS-CoV-2) and causes a disease called coronavirus disease-19 (COVID-19) [1–3]. Infection with SARS-CoV-2 mainly causes respiratory tract infections ranging from asymptomatic and mild disease forms to severe disease and death [4].

Severe acute respiratory syndrome coronavirus 2 is the 7th novel highly pathogenic human beta coronavirus that causes lower respiratory tract illness [5, 6]. Apart from the asymptomatic patients, cough, difficulty in breathing, fatigue, and fever are the main clinical manifestations of COVID-19. But symptoms of diarrhea, expectoration, and headache seemed unusual. According to the radiographic evidence, around fifty percent of patients with COVID-19 have developed severe pneumonia. Due to acute respiratory distress syndrome (ARDS) or multiple organ failure, almost 1/3 of the patients need intensive care [1, 7].

People with renal disease or other comorbidities that can lead to severe medical conditions such as pulmonary disease, diabetes, chronic heart failure, and hypertension and older adults and people with weak immunity are at high risk for developing severe COVID-19 disease [8–10]. The patients with COVID-19 and association with abnormal hematological results were indicated according to some previous studies [11, 12]. Urine dry biochemical tests are used as the economical, convenient, and quick method for the auxiliary diagnosis and monitoring of treatment effects of kidney diseases or complications due to another severe illness [13, 14].

In low- and middle-income countries, infectious diseases, most notably, parasitic infections, affect millions of people with high prevalence rates [15, 16]. Highly complex and multicellular parasites as well as unicellular organisms are among the most important organisms that contribute to the global disease burden associated with intestinal parasitic infection. However, the distribution of parasitic infections differs extensively in the different regions of the world [15, 17]. Such tenacious and chronic parasitic infections have been revealed to alter the clinical outcomes of other infections through direct host immune response modulation [18]. Previously existing infections with parasites amend the host's body defenses to infection with SARS-CoV-2, with postulated detrimental and beneficial effects in some geographic areas [16, 19].

The number of COVID-19 cases and fatalities is increasing throughout Ethiopia [20]. Studying the clinical profiles of patients with COVID-19 helps clinicians easily identify suspects from various backgrounds, determine targeted therapy, and improve patient management. In addition, determining the laboratory profiles of patients is critically important to evaluate disease severity, the efficacy of treatments, immunopathology, prognosis, and recovery of patients. However, there is limited research data on the current theme in Ethiopia, particularly in the northwest part. This study is, therefore, aimed at determining the association of COVID-19 disease severity with intestinal parasite coinfection and urine biochemical parameters among confirmed COVID-19 patients admitted at Debre Markos COVID-19 Center.

## 2. Materials and Methods

### 2.1. Study Area, Design, and Period

This prospective cohort study was conducted among COVID-19-confirmed patients at Debre Markos University COVID-19 treatment center in Debre Markos town from January 1, 2021, to March 30, 2021. Debre Markos town is located northwest of Addis Ababa, the capital of Ethiopia, at 300-kilometer distance and 265 kilometers away from Bahir Dar, the capital of Amhara National Regional State. The town has one COVID-19 isolation and treatment center established by Debre Markos University with the capacity to test, treat, and monitor COVID-19 patients.

### 2.2. Study Population

All RT-qPCR-confirmed new COVID-19 patients admitted to Debre Markos University COVID-19 Center during the study period were our study population. COVID-19 patients who have been under treatment for COVID-19 symptoms and parasite infections and history of previous COVID-19 infections were not eligible for this study.

### 2.3. Sample Size Calculation and Sampling Technique

In Ethiopia, there were no similar studies published about the association of COVID-19 disease severity with intestinal parasite coinfection and urine biochemical parameters. Thus, the sample size was determined using the formula for single population proportion by considering assumptions of 50% prevalence, a 5% margin of error, and a 95% confidence level.

(1) $$\begin{array}{c} n=\frac{{\left(Za/2\right)}^{2}*p\left(1-P\right)}{d^{2}},\\ n=\frac{{\left(1.96\right)}^{2}*0.5\left(1-0.5\right)}{{\left(0.05\right)}^{2}}. \end{array}$$

The calculated total sample size was 384, but the number of confirmed cases of COVID-19 in the last three months before our study in the COVID-19 treatment center was 180, which is less than 10,000 and needs a population correction formula. Thus, using the correction formula, the corrected sample size was 123, and by considering a 10% nonresponse rate, the final sample size was 136. The study participants were selected consecutively during admission.

### 2.4. Data Collection

Six trained data collectors, two laboratory personnel, two clinical nurses, and two physicians, participated in the data collection. Patient's sociodemographic, behavioral, and clinical characteristics such as age, sex, residence, marital status, occupation, education level, smoking, alcohol drinking, traditional medicine use, body mass index, blood pressure, sign symptom of COVID-19 disease, and comorbidity were collected by using a predesigned structured questionnaire. The questionnaire was adopted and prepared by using the International Severe Acute Respiratory and Emerging Infection Consortium's (ISARIC) CRFs for emerging severe acute respiratory infection guidelines [21].

### 2.5. Specimen Collection and Laboratory Analysis

Before any data collection, every participant was confirmed for SARS-CoV-2 infection using RT-qPCR by taking nasopharyngeal and throat swab specimens according to the WHO guideline recommendations (DaAn Gene kit; China). About 10 to 15 ml of freshly voided midstream urine sample was collected in a clean screw-capped, wide-mouth container from each patient. The urine sample for critical COVID-19 patients, who cannot provide a specimen by themselves, is collected directly from the catheter. Then, the urine specimen was tested for the urine biochemical parameters such as leukocyte esterase, protein, blood, ketone, glucose, bilirubin, urobilinogen, and nitrate using a dry urine dipstick kit (Combur-Test strip, Roche Diagnostics, India). Also, fresh stool specimens were obtained for direct saline wet mount microscopic examination for detection of parasites (ova, cyst, oocysts, larva, or trophozoite) and further confirmed by using the formalin-ether concentration method to increase sensitivity and better detection of parasite ova.

### 2.6. Data Quality Assurance

To assure the quality of data, the data collection tool was pretested before actual data collection and the data collectors were well trained. The laboratory analyses were carried out based on standard operating procedures (SOPs), and quality control was performed for each test using known positive and negative samples. In addition, the manufacturer's instructions were strictly followed during reagent storage, preparation and testing, and result interpretation.

### 2.7. Variable Measurement and Outcomes

The proportion of COVID-19 disease severity among SARS-CoV-2-positive patients with and without abnormal urine biochemical parameters and the intestinal parasitic coinfection was the main outcome of this study. Based on the symptoms of COVID-19 cases, the severity of the disease was classified as asymptomatic, mild/moderate, severe, and critical cases. This disease clinical severity classification was made according to the Diagnosis and Treatment Program of 2019 New Coronavirus Pneumonia (sixth trial version) [22]. Furthermore, the patients were followed up until discharged after recovery or death.

### 2.8. Data Management, Processing, and Analysis

Data was entered, coded, and cleaned using EpiData software version 3.1 applications. Then, cleaned data was exported to SPSS software version 24.0 for statistical analysis. The sociodemographic and clinical characteristics of the study participants were summarized by using descriptive statistics. The findings were presented by using tables and graphs, and the main features were narrated. The association between urine biochemical result and intestinal parasite coinfection with SARS-CoV-2 and COVID-19 clinical severity was determined by using ordinal logistic regression analysis. Additionally, the chi-square test or Fisher exact test was computed to assess association with outcome variables as necessary. Finally, a *P* value < 0.05 was used as a cutoff point for the presence of statistical significance.

## 3. Results

### 3.1. Sociodemographic and Clinical Characteristics

A total of 136 laboratory-confirmed SARS-CoV-2-infected patients were enrolled in this study. The participant's median age was 48 years ranging from 16 to 78 years. Among them, 67 (49.3%) were 40-59 years old, 36 (26.5%) were 20-39 years old, and 31 (22.8%) were 60-79 years old. Among the participants in this study, the youngest and oldest patients were 16 years and 78 years old, respectively (Table 1). The majority (86 (62.5%)) of the participants were men. Of the 136 cases, 39 (28.7%) had died and the remaining recovered from COVID-19 disease. The most common comorbidity among COVID-19 patients was cardiac heart failure (42 (30.9%)), hypertension (28 (20.6%)), diabetes (17 (12.5%)), and chronic obstructive pulmonary disease (15 (11.0%)).

### 3.2. Clinical Signs and Symptoms of COVID-19 Patients

Most patients were admitted to the treatment center with joint pain (130 (95.6%)), dry cough (128 (94.1%)), loss of appetite (127 (93.4%)), and headache and vomiting (127 (93.4%)) (Table 2).

### 3.3. Urine Biochemical Parameters of COVID-19 Patients

Among 136 patients tested for urine biochemical parameters during admission, 104 (76.5%) have abnormal urine analysis results (which means an outcome that deviates from the normal range of result/value established by the manufacturer). Male patients account for 64 (61.5%) of this abnormal urine biochemical test result compared to female (40 (38.5%)). From the abnormal urine biochemical tests observed, the urine blood test was positive for 54 (39.7%) patients. Thirty-six (26.5%) patients showed positive glucose results. Among the glucose-positive patients, over half of the patients (21 (58.3%)) were +1 followed by +2 (15 (41.7%)), +3, and +4 which accounts for 4 (11.1%) each. Thirty (21.1%) patients were positive for ketone, and none of the patients were positive for urobilinogen (Table 3). Among the critical COVID-19 patients, 80.6% showed abnormal urine biochemical parameters. From this abnormal urine biochemical parameters determined among critical COVID-19 patients, protein, blood, and glucose account for the highest proportions (80.6%, 48.3%, and 45.2%, respectively). Even if death is high among COVID-19 patients with abnormal urine biochemical test results, there was statistically no significant difference (*P* > 0.05). The differences in the positive rates of glucose, protein, blood, and leukocyte esterase were significantly higher in patients with critical COVID-19 disease compared to mild and severe COVID-19 disease (*P* < 0.05). However, there is no statistically significant difference in the detection rates of ketone, bilirubin, and nitrate among the different COVID-19 disease severities (*P* > 005).

### 3.4. Intestinal Parasite Coinfection among COVID-19 Patients

Among the 136 COVID-19 patients who were examined for intestinal parasitic infections, from stool specimens, 22 (16.2%) were parasite-infected. The highest prevalence of 14/58 (24.1%) of intestinal parasitic infection was observed among COVID-19 patients with mild disease clinical symptoms. The lowest frequency of intestinal parasite infection (3 (9.7%)) was observed among patients with critical COVID-19 disease (Table 4). Of the intestinal parasite infections, helminth and protozoa comprised 86.4% and 13.6%, respectively. *Giardia lamblia* and *Entamoeba histolytica* were the only protozoan infections found, and *Ascaris lumbricoid*, *Hookworm*, and *Trichuris trichiura* were the most common helminth infection. Among the COVID-19/intestinal parasite-coinfected patients, more than half (54.5%) had polyparasitism, which means infestation with two or more parasite species (Table 5).

The clinical outcome (death) among COVID-19 patients was 39 (28.7%). Among them, the highest death rate of 26 (83.8%) was observed among critical patients with the lowest (3 (9.7%)) intestinal parasite COVID-19 coinfections (Table 4).

To test the association between intestinal parasite infection and other clinical factors with COVID-19 disease severity, ordinal logistic regression analysis was used. In univariate analysis, age group, history of smoking, having at least one comorbidity, hypertension, diabetes, cardiac heart failure, chronic liver disease, and chronic obstructive pulmonary disease were all associated with increased odds of having severe COVID-19 disease. But, in multivariate analysis, only being in the age group > 60 years old, having at least one comorbidity, hypertension, diabetes, cardiac heart failure, and chronic obstructive pulmonary disease were significantly associated with increased odds of severe COVID-19 disease (*P* < 0.05) (Table 6). By adjusting age group and comorbidities, patients with COVID-19 coinfected with any intestinal parasites had lower odds of developing severe COVID-19 compared to patients without intestinal parasite infection, with an adjusted odds ratio (AOR = 0.37; 95% CI 0.147-0.944; *P* = 0.037). Moreover, COVID-19 patients who had helminth coinfection (AOR = 0.475; 95% CI 0.243-0.531; *P* < 0.001) and multiple parasite infections (AOR = 0.435; 95% CI 0.243-0.721; *P* < 0.001) had a lower odds of developing severe COVID-19 disease compared to COVID-19 patients without intestinal parasite infection (Table 6).

## 4. Discussion

The worldwide spread outbreak of the COVID-19 pandemic has led to huge economic losses and millions of patient deaths worldwide. Since then, COVID-19 was announced by the WHO as the 6th public health emergency of international concern. The disease onset of some COVID-19 patients has shown rapid progression to multiple organ failure, and older male patients with the comorbid disease are more likely to have respiratory failure, and many die due to critical COVID-19 [23]. This study also showed similar findings in which patients with age older than 60 years are more prone to have severe COVID-19 disease than patients younger than age 60 years (*P* = 0.006). Moreover, this finding is consistent with other study reports that showed increased risks of developing severe COVID-19 disease among older age groups [24–27]. Even if, sociodemographic characteristics like sex, residence, and educational status are significantly associated with the severity of COVID-19 disease in different studies, and our study is in contrast with their finding [24]. The difference in study reports might be due to differences in geography, population, and sample size.

In this study, patients who had comorbidity, particularly hypertension, diabetes, chronic pulmonary obstructive disease, and cardiac heart failure, had an increased risk of developing severe COVID-19 compared to patients presented with no comorbidity. This finding is consistent with studies reported from different parts of the world in which comorbidity was significantly associated with the increased severity of COVID-19 [25–27].

This study showed statistically significant differences in the positivity to glucose, blood, and leukocyte esterase among the different disease clinical severities of COVID-19 (*P* < 0.05). Our findings indicate that SARS-CoV-2 infection leads to kidney damage in patients, as evidenced by abnormal urine biochemical findings, such as protein, blood, and leukocyte. Our study finding is supported by different study reports from China [28, 29]. There was a significant increase in positive rates of glucose, protein, blood, and leukocytes among the severe and critical groups, compared with mild COVID-19 patients. This finding is similar to a previous study report from China [29] in which glucose and protein were significantly raised among the severe and critical groups. Nevertheless, leukocyte and blood showed no statistically significant difference among the different clinical disease severities. Such differences in findings between studies might be due to differences in study population and comorbidity among patients. The differences in the detection rates of ketone, bilirubin, and nitrate did not show statistically significant differences among the different clinical disease severities of COVID-19 in our study (*P* > 0.05). This result is supported by study findings reported from another study [29]. This indicates that the impermanent increase in glucose (hyperglycemia) will not cause the patient to develop ketoacidosis which indirectly shows that the patient's disease severity was primarily because of abnormal respiratory function and other comorbidities.

In our study, 16.2% of the COVID-19 patients were found coinfected with intestinal parasites during admission to the center. Among them, the highest prevalence (24.1%) of intestinal parasitic infection was observed among COVID-19 patients who presented with mild disease clinical severity. This finding is lower than the study finding of 37.8% from Mekele [24]. This discrepancy in the prevalence of intestinal parasite coinfection might be a small sample size in our study and the difference in population. In this study, COVID-19 patients who have intestinal parasite coinfection had lower odds of developing clinically severe COVID-19 compared to noninfected (AOR = 0.37; 95% CI = 0.147 − 0.944; *P* = 0.037). Similarly, COVID-19 patients who had helminth coinfection and multiple parasite infections had lower odds of developing severe COVID-19 disease compared to COVID-19 patients without intestinal parasite infection in our study. This might be due to infections with intestinal parasites (helminthes), which are known to modulate responses associated with inflammation and induce an immunotolerogenic state in the host. As a result of that, COVID-19-induced inflammation will be modulated with the help of helminth-induced immunoregulation, and thus, the interaction of intestinal parasite (helminthes) and COVID-19 coinfections may perhaps be helpful for the patients [24, 30–32]. Our study finding is also supported by a study finding reported from another part of Ethiopia stating “COVID-19 was less severe in those patients who had pre-existing intestinal parasite co-infections” [24].

In our study, the death rate was higher among COVID-19 patients without intestinal parasite infection than COVID-19/intestinal parasite-coinfected patients. Our finding is strongly supported by different studies and reports that showed a low fatality rate of COVID-19 disease in lower-middle-income country settings where intestinal parasitic infections are endemic [32–35].

### 4.1. Limitations of the Study

In this study, we did not use the gold standard technique, Kato-Katz, for stool examination, which is highly sensitive and used to assess the infection intensity of parasites due to the unavailability of kits in the market during the pandemic.

## 5. Conclusions

Our study demonstrates that routine urine biochemical tests are useful in the evaluation of the disease severity in patients with COVID-19. Being older age and having comorbidity were associated with the severity of COVID-19. Patients with intestinal parasitic infection demonstrated mild COVID-19 disease severity. Therefore, conducting regular urine biochemical analyses for the monitoring and evaluation of COVID-19 progress and further studies on the effect of the interaction among parasite-microbiome on COVID-19 clinical severity and its mechanisms proposes possibilities for novel therapeutic and preventive interventions.

## Figures and Tables

**Table 1:** Sociodemographic and clinical characteristics of 136 COVID-19-confirmed patients without and with intestinal parasite infections, in Northwest Ethiopia.

Patients characteristics	Total patients (*n* = 136)	Without intestinal parasite infection (*n* = 114)	With intestinal parasite infection (*n* = 22)	*x* ^2^ *P* value
Age group				
<20	2 (1.47%)	2 (1.80%)	0 (0.0%)	0.008
20-39	36 (26.5%)	33 (28.9%)	3 (16.6%)	
40-59	67 (49.3%)	49 (43.0%)	18 (81.8%)	
60-79	31 (22.8%)	30 (26.3%)	1 (4.5%)	

Sex				
Female	51 (37.5%)	44 (38.6%)	7 (31.8%)	0.635
Male	85 (62.5%)	70 (61.4%)	15 (68.2%)	

Residence				
Urban	111 (81.6%)	96 (84.2%)	15 (68.1%)	0.021
Rural	25 (18.4%)	18 (15.8%)	7 (31.8%)	

Occupations				
Employed	39 (28.7%)	33 (28.9%)	6 (27.3%)	0.621
Unemployed	57 (41.9%)	45 (39.5%)	12 (54.5%)	
Student	26 (19.1%)	23 (20.2%)	3 (13.6%)	
Health professionals	14 (10.3%)	13 (11.4%)	1 (4.5%)	

History of smoking	20 (14.7%)	15 (13.1%)	5 (22.7%)	0.320
Nonsmokers	116 (85.3%)	101 (86.9%)	17 (77.3%)	

Alcohol drinking	132 (97.0%)	110 (96.5%)	22 (100%)	0.912
Nondrinker	4 (3.0%)	4 (3.5%)	0 (0.0%)	

Traditional medicine use	38 (27.9%)	32 (28.1%)	6 (27.3%)	0.939

Comorbidity				
At least 1 comorbidity	76 (55.9%)	58 (50.9%)	18 (81.8%)	0.009
Hypertension	28 (20.6%)	28 (24.6%)	0 (0.0%)	0.007
Diabetes	17 (12.5%)	17 (14.9%)	0 (0.0%)	0.074
HIV	4 (2.9%)	4 (3.5%)	0 (0.0%)	0.489
Malignant tumor	2 (1.5%)	2 (1.8%)	0 (0.05)	0.702
Asthma	11 (8.1%)	8 (7.0%)	3 (13.6%)	0.385
COPD	15 (11.0%)	13 (11.4%)	2 (9.1%)	0.549
CKD	4 (2.9%)	4 (3.5%)	0 (0.0%)	0.489
CLD	8 (5.9%)	8 (7.0%)	0 (0.0%)	0.354
CHF	42 (30.9%)	40 (35.1%)	2 (9.1%)	0.021

Clinical outcome				
Death	39 (28.7%)	34 (29.8%)	5 (22.7%)	0.612
Recovered	97 (71.3%)	80 (70.2%)	17 (77.3%)	

HIV: human immunodeficiency virus; COPD: chronic obstructive pulmonary disease; CKD: chronic kidney disease; CLD: chronic liver disease; CHF: cardiac heart failure. The calculated *P* values are from the chi-square test or Fisher exact test for categorical variables as appropriate.

**Table 2:** Signs and symptoms observed among 136 COVID-19-confirmed patients in Northwest Ethiopia.

Clinical symptoms	Frequency (%)
Fever	93 (68.4%)
Dry cough	128 (94.1%)
Shortness of breath	100 (73.5%)
Headache and vomiting	127 (93.4%)
Joint pain	130 (95.6%)
Loss of appetite	127 (93.4%)
Chest pain	65 (47.8%)
Sore throat	112 (82.4%)
Loss of taste and/or smell	45 (33.1%)

**Table 3:** Urine biochemical parameters of 136 COVID-19-confirmed patients and severity of the disease, Northwest Ethiopia.

Urine biochemical parameters	Total (%)	Mild (*n* = 58)	Severe (*n* = 47)	Critical (*n* = 31)	*P* value
Normal biochemical results	32 (23.5)	11 (19.0)	15 (31.9)	6 (19.4)	0.124
Abnormal biochemical results	104 (76.5)	47 (81.0)	32 (68.1)	25 (80.6)	0.246
Leukocyte esterase	7 (5.9)	3 (5.2)	0 (0.0)	5 (16.1)	0.002
Protein	35 (25.7)	7 (12.06)	10 (13.5)	18 (80.6)	0.012
Blood	54 (39.7)	27 (46.5)	12 (16.2)	15 (48.3)	0.007
Ketone	30 (21.1)	10 (17.2)	9 (12.2)	11 (35.5)	0.069
Glucose	36 (26.5)	10 (17.2)	12 (16.2)	14 (45.2)	0.037
Bilirubin	6 (4.4)	3 (5.2)	0 (0.0)	3 (9.6)	0.05
Urobilinogen	0 (0)	0 (0.0)	0 (0.0)	0 (0.0)	—
Nitrate	2 (1.5)	0 (0.0)	2 (2.7)	0 (0.0)	0.171

**Table 4:** Intestinal parasite infection and COVID-19 disease severity, Northwest Ethiopia.

Disease severity	Total (%)	Patients infected with parasites (%)	Death (%)	AOR (95% CI)	*P* value
Mild/moderate	58 (42.6)	14 (24.1)	1 (1.7)	0.37 (0.15-0.94)	0.037
Severe	47 (34.6)	5 (10.6)	12 (25.5)		
Critical	31 (22.8)	3 (9.7)	26 (83.8)		
Total number (%)	136 (100)	22 (16.2)	39 (28.7)		

**Table 5:** The proportion of identified intestinal parasites among COVID-19 patients.

Identified parasites (*n* = 22)	Frequency (%)
Helminthes (*A. lumbricoid*, *H. worm*, and *T. trichiura*)	19 (86.4)
Protozoa (*G. lamblia* and *E. histolytica*)	3 (13.6)
Polyparasitism (any)	12 (54.5)
Helminthes and protozoa	2 (9.1)
Helminth plus helminth	10 (45.4)

**Table 6:** Associated factors with severe COVID-19 disease among confirmed 136 patients.

Patients characteristics	Univariate analysis		Multivariate analysis	
	COR (95% CI)	*P* value	AOR (95% CI)	*P* value
Age group				
>60	0.714 (0.267-1.161)	0.002	1.821 (1.224-9.421)	0.006
<60	1			

Sex				
Female	0.531 (0.123-1.186)	0.312	—	—
Male	1			

Residence				
Urban	0.452 (0.621-1.524)	0.742	—	—
Rural	1			

Occupations				
Unemployed	1		—	—
Employed	0.421 (0.145-1.721)	0.2742	—	—
Student	0.805 (0.621-2.121)	0.642	—	—
Health professionals	1.021 (0.112-1.431)	0.941	—	—

History of smoking	1.183 (0.217-2.148)	0.016	1.225 (0.355-4.230)	0.748
Nonsmoker	1			

Alcohol drinking	0.880 (0.134-2.895)	0.392	—	—

Body mass index				
18.5-24.9	1			
<18.5	0.721 (0.213-1.841)	0.621		
25.0-29.9	0.532 (0.0221-2.321)	0.714		
>30.0	0.354 (0.919-1.801)	0.921		

Traditional medicine use	0.088 (0.657-1.832)	0.817	—	—

Comorbidity				
At least 1 comorbidity	1.269 (1.360-9.898)	0.001	2.912 (1.542-7.245)	0.001
Hypertension	2.181 (1.310-3.051)	<0.001	6.543 (1.999-21.411)	0.002
Diabetes	3.918 (2.344-5.492)	<0.001	71.494 (11.744-44.081)	<0.001
HIV	0.602 (0.542-1.338)	0.543	—	—
Malignant tumor	0.472 (0.096-3.040)	0.718	—	—
Asthma	0.507 (0.629-1.644)	0.382	—	—
COPD	1.897 (1.816-2.978)	0.001	9.001 (1.758-4.073)	0.008
CKD	1.570 (0.378-2.767)	0.114	—	—
CLD	0.605 (1.043-2.791)	0.043	3.283 (0.499-21.58)	0.216
CHF	1.465 (1.622-2.081)	<0.001	3.577 (1.425-8.981)	0.007

Positive urine biochemical test	0.173 (0.561-1.907)	0.644	—	—

Parasite infection (any)	0.967 (0.532-0.864)	0.008	0.37 (0.151-0.941)	0.037
Helminth infection	0.376 (0.213-0.612)	<0.001	0.475 (0.243-0.531)	0.001
Protozoa infection	0.621 (0.235-1.435)	0.521	—	—
Polyparasitism	0.331 (0.124-0.625)	0.001	0.435 (0.243-0.721)	0.001

HIV: human immunodeficiency virus; COPD: chronic obstructive pulmonary disease; CKD: chronic kidney disease; CLD: chronic liver disease; CHF: cardiac heart failure.

## Data Availability

Research data can be presented upon a reasonable request.

## Abbreviations

COVID-19: Coronavirus disease 2019
SARS-CoV-2: Severe acute respiratory syndrome coronavirus 2.
